# Effectiveness of an Acupuncture Steam-Warming Eye Mask on Dry Eye Disease in Visual Display Terminal Users: A Prospective Randomized Controlled Trial

**DOI:** 10.3390/diseases12080192

**Published:** 2024-08-22

**Authors:** Chia-Yi Lee, Shun-Fa Yang, Ching-Hsi Hsiao, Chi-Chin Sun, Chao-Kai Chang, Jing-Yang Huang, Yih-Shiou Hwang

**Affiliations:** 1 Institute of Medicine, Chung Shan Medical University, Taichung 40201, Taiwan; 2 Nobel Eye Institute, Taipei 10041, Taiwan; 3 Department of Ophthalmology, Jen-Ai Hospital Dali Branch, Taichung 41265, Taiwan; 4 Department of Medical Research, Chung Shan Medical University Hospital, Taichung 40201, Taiwan; 5 Department of Ophthalmology, Chang Gung Memorial Hospital, Taoyuan 33305, Taiwan; 6 Department of Chinese Medicine, Chang Gung University, Taoyuan 33302, Taiwan; 7 Department of Ophthalmology, Chang Gung Memorial Hospital, Keelung 20448, Taiwan; 8 Department of Optometry, Da-Yeh University, Chunghua 51591, Taiwan; 9 Department of Medicine, Chang Gung University, Taoyuan 33302, Taiwan

**Keywords:** acupuncture, visual display terminal, steam-warming eye mask, dry eye disease, meibomian gland dysfunction

## Abstract

We aim to evaluate the effectiveness of an acupuncture steam-warming eye mask (ASEM) on dry eye disease (DED) in visual display terminal (VDT) users. This prospective randomized clinical trial included VDT users with DED-related features who were randomly assigned to the ASEM group (ASEM for 2 weeks, 20 participants) or the steam-warming eye mask (SEM) group (SEM for 2 weeks, 20 participants). The tear film break-up time (TBUT), Schirmer test, tear meniscus height, ocular surface staining scores, eyelid and meibomian gland exam, subjective symptoms, and quality of life (QoL) scores before and after treatment were collected. A generalized linear mixed model was applied to compare the improvement of symptoms and signs between the two groups. After the 2-week treatment, all the subjective symptoms and questionnaire scores in the ASEM group improved significantly (all *p* < 0.05), whereas the feelings of relaxation, comfortable, and refreshment did not change in the SEM group (both *p* > 0.05). The TBUT, tear meniscus height, and meibum quality in the lower eyelid were significantly better in the ASEM group than the SEM group (all *p* < 0.05), whereas no significant changes were observed in the Schirmer test and ocular surface staining scores. Compared with the SEM group, the ASEM group experienced a stronger feeling of refreshment (*p* = 0.013), lower sensation of ocular discharge (*p* = 0.031), higher TBUT (*p* = 0.045), better meibomian gland expressibility of both eyelids (both *p* < 0.05), and better meibum quality of both eyelids (both *p* < 0.05), even after adjustments for age and sex. In conclusion, comparing with SEM, ASEM can improve some subjective DED symptoms, tear film stability, and meibum status in VDT users.

## 1. Introduction

Visual display terminals (VDTs), including computers, smartphones, and tablets, have become an essential part of daily life [1]. Approximately one billion personal computers are in use globally [2], and 95% of individuals aged 18–34 years old use smartphones [3]. However, VDTs exert negative effects on the human body, including dermatological and musculoskeletal disorders, such as rosacea, shoulder pain, cervical stiffness, and temporomandibular disorder, even among relatively light users [4,5,6]. Psychological comorbidities, such as poor mental health status, anxiety, and insomnia, may develop in VDT users [7,8,9]. Moderate VDT use can cause ocular complications, such as accommodation defects, comitant esotropia, and dry eye disease (DED) [10,11,12,13]. 

DED is an inflammatory disease with reduced tear film amount and quality. Common DED symptoms include dryness, grittiness, foreign body sensation, discharge, soreness, fatigue, intermittent blurry vision and unspecific ocular discomfort [14]. DED in most VDT users is characterized by a moderate-to-severe ocular surface disease index, high tear osmolarity, lacrimal hypofunction, epithelial defects of the cornea and conjunctiva, and meibomian gland dysfunction (MGD) [2,13,14,15,16]. In a recent study, impaired blinking patterns and change in parasympathetic signaling were proposed as a pathophysiology of DED in VDT users [17]. Osaka et al. reported that ≥60% of VDT users had DED with a decreasing tear film break-up time (TBUT) [14] and that MGD developed in >20% of VDT users and decreasing TBUT and MGD would imply evaporative DED [14]. Also, the ocular surface damage and irritation resulting from DED significantly impair the quality of life (QoL) [18,19]. Although increment of blink rate, methods to prevent ocular inflammation, and ergonomic training have all been proposed to prevent DED associated with VDT usage, there is no consensus on best treatment [20]. Recently, the application of autologous serum, allogeneic serum, and umbilical cord serum showed fair effectiveness on treating severe DED [21], while their effects on VDT users remained unclear. Accordingly, developing effective and convenient management of DED for VDT users is warranted.

A steam-based eyelid-warming device that can melt meibomian gland lipids had been used for MGD treatment for decades; moreover, it can retain heat more effectively than a warmed facecloth [22,23,24]. This device significantly improved TBUT and Schirmer test scores in patients with MGD [25,26] and significantly improved TBUT and tear meniscus volume in those with DED [27]. A study demonstrated that the application of this steam-warming eye mask (SEM) could improve tear film stability and QoL in VDT users with evaporative DED [28]. Given that acupuncture had also been proposed as an effective treatment for DED [29,30,31], we speculated that the use of the SEM combined with acupuncture may be highly effective for treating DED in VDT users.

We investigated the effectiveness of an acupuncture steam-warming eye mask (ASEM), which was designed with acupuncture function, on DED management and compared it with SEM in terms of tear film stability, tear film secretion, subjective symptoms, ocular surface health, eyelid condition, and QoL.

## 2. Materials and Methods

### 2.1. Ethical Declaration

Our study adhered to the tenets of the 1964 Declaration of Helsinki and its later amendments, and the study protocol was approved by the Institutional Review Board of Chang Gung Memorial Hospital (project code: 201801881B0A3, date of approval: 22 April 2020). Also, our study was registered in the ClinicalTrials.gov (registration number: NCT04584216). Written informed consent was obtained from all participants after the study coordinator thoroughly explained the study aims and procedures to them.

### 2.2. Study Design and Participants

A prospective randomized clinical trial (registration number: NCT04584216) was conducted at Linkou Chang Gung Memorial Hospital, a tertiary hospital in northern Taiwan. Volunteers fulfilling the following criteria were recruited: (1) at least one of eight DED symptoms that were experienced “often” or “constantly”: tired eyes, dry eyes, ocular discomfort, blurred vision, gritty eyes, lack of relaxing feeling, lack of comfortable feeling, and lack of refreshing feeling, which were modified from the questionnaire developed by Toda et al. [32], (2) tear film instability with decreased TBUT < 10 s in our ophthalmic clinic, and (3) using VDTs for ≥6 h a day for >3 months. We recorded the total time (being broken up) of VDT usage from our participants during inquiry of initial assessment. We excluded patients with prominent ocular abnormality, including: (1) excessive meibomian lipid dysfunction with the presence of toothpaste meibum substances in more than half of any eyelid; (2) eye abnormalities, such as allergic disease, infectious conjunctivitis, and autoimmune disease; (3) periocular skin abnormalities, such as rash or eczema; (4) hypersensitivity to heating; and (5) treated with any DED managements including, but not limit to, artificial tear, overnight ointment, steroid, immunosuppressant, and intense pulse light therapy within one year before the recruitment. Participants were then randomized into the ASEM or SEM groups (N = 20 in each group) by drawing lots.

### 2.3. Treatment with Different Steam-Warming Eye Mask

**Figure 1:**
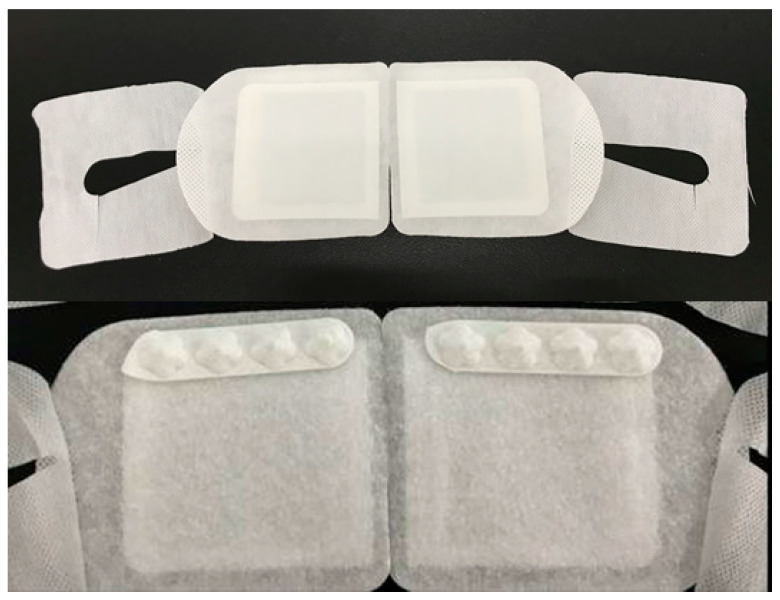
The front (**upper**) and back (**lower**) appearance of the acupuncture steam-warming eye mask.

**Figure 2:**
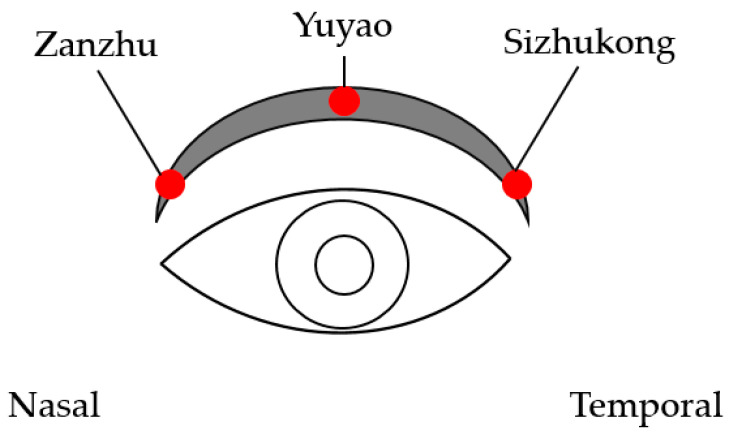
The diagram of the three acupoints.

Two interventions (ASEM and SEM) were applied in this study. The SEM (Megrhythm, Kao Corporation, Chuo-ku, Tokyo, Japan) is a disposable eye mask containing iron and water in a sealed heating unit, and it generates moist heat (40 °C) through the oxidative reaction of iron, with the heat being applied for roughly 15–20 min per session. The ASEM is not an electric acupuncture device and cannot be plugged into an electric socket; thus, the safety may be adequate. The design of the ASEM is identical to the SEM but with additional protrusions of the acupoints’ calls Cuanzhu (computing compatible number: BL2), Yuyao (computing compatible number: EX_HN4), and Sizhukong (computing compatible number: TE23) that are located around the upper orbital rim. The appearance of ASEM is presented in Figure 1, and we chose the three acupoints due to their effectiveness for treating DED in a previous study [33]. The picture of the three acupoints is presented in Figure 2. The back side of the ASEM has 4 points to ensure that the patients can massage onto the three acupoints we selected. The patients press the acupoint from the nasal side to the temporal side, and each acupoint is pressed for 15 s. The whole massage course was repeated three times. The ASEM/SEM is disposable, and each participant used a new one every day. Participants were randomized to either the ASEM group or the SEM-only group, with each group applying the respective device for 20 min daily for 2 h from 17:00 to 19:00. We decided on the 20 min usage since the 15 min usage from previous study is effective [28]. None of the participants had used massage acupuncture before. Regarding the instruction of ASEM/SEM, the ophthalmologist demonstrated the wearing of the ASEM/SEM, the procedure that releases the moist heat, the sites participants need to press for the acupuncture effect, and the position the participant should be in during the usage of ASEM/SEM (i.e., sitting position). The complete instruction time required 10–20 min for each participant until they fully understood the application of ASEM/SEM. At 2 weeks after the initial examination (Day 15), the participants visited the clinic for an evaluation of the effectiveness of SEM and ASEM. The physician was blinded to groupings while examining the participants.

### 2.4. Primary Outcomes

The primary outcomes included the TBUT, Schirmer I test, corneal vital staining, conjunctival vital staining, and meibomian gland condition assessment, as described previously [28]. The ophthalmic examinations were performed on Days 1 and 15. The TBUT exam was performed under a slit lamp by using a fluorescein dye, and the time between the blink and the first dark spot was measured using a stopwatch. For the Schirmer I test, the Schirmer strip is applied, and the patients close their eyes for 5 min; the Schirmer strip is then removed, and the length of the wet portion is recorded. Corneal and conjunctival epithelial damage in three areas (the cornea, nasal conjunctiva, and temporal conjunctiva) is examined using fluorescein dye and scored from 0 (i.e., no damage) to 3 (i.e., widespread loss of epithelium), with total scores ranging from 0 to 9, as described previously [34]. The meibum quality of the left eye is graded as follows: 0 (clear and easily expressed), 1 (cloudy and easily expressed), 2 (cloudy and expressed with moderate pressure), and 3 (not expressed even with strong pressure) [35]. For safety assessment, pretreatment and posttreatment intraocular pressure (IOP) and uncorrected visual acuity (UCVA) were also recorded.

### 2.5. Secondary Outcomes

The secondary outcomes include the subjective symptoms and QoL according to several questionnaires. The participants were asked to complete several DED questionnaires to assess their subjective DED symptoms on Days 1 and 15 to evaluate all the possible DED-related symptoms as detailed as possible. The basic questionnaire we used for participant screening, which was modified from the questionnaire developed by Toda et al. [32], was also administered. However, we replaced the four answers (i.e., never, sometimes, often, and constantly) of the basic questionnaire by a visual analog scale ranging from 0 to 100. Moreover, we applied the computer vision syndrome questionnaire (CVSQ), which contains 16 questions evaluating the frequency and intensity of DED-related symptoms, to our VDT users [36]. To evaluate the overall DED-associated QoL, we used the Dry Eye-Related Quality of Life Score (DEQS) developed by the Japanese Dry Eye Society [37]. This questionnaire consists of 15 DED-related questions, with total scores ranging from 0 to 100.

### 2.6. Statistical Analysis

All the statistical analyses in our study were performed using SAS version 9.4 (SAS Institute Cary, NC, USA). The statistical power of the current study was 0.77 with a 0.05 alpha value and a medium effect size, which was generated using G∗power version 3.1.9.2 (Heinrich Heine Universität at Düsseldorf, Germany). The data were presented as means with standard deviation (SD), and all the outcomes in this study were normally distributed according to the results of the Kolmogorov–Smirnov test (all *p* > 0.05). Between-group comparisons of treatment effectiveness were performed using the Wilcoxon signed-rank test. For intergroup comparison of the same time period, the Mann–Whitney U test was used for continuous variables, and Fisher’s exact test was used for ordinal variables. Between-group comparison of DED improvement after treatment was performed using a generalized linear mixed model to assess the subjective symptoms, ocular discharge, TBUT, Schirmer test, tear meniscus height, ocular surface staining score, meibomian gland expressibility, and meibum quality. Further, we considered age and sex as fixed effects in the generalized linear mixed model to adjust their influence on DED. A *p* < 0.05 was considered statistically significant.

## 3. Results

### 3.1. Baseline Characters of Study Population

The patients’ baseline characteristics are summarized in Table 1. The mean age was 34.17 ± 10.23 years and 35.22 ± 11.41 years in the ASEM and SEM groups, respectively, and the male-to-female ratio was 1:2 and 1:4, respectively (both *p* > 0.05). DED-related parameters, including the TBUT, Schirmer test, tear meniscus height, ocular surface staining score, and meibum quality score, were also comparable between the two groups (all *p* > 0.05; Table 1).

### 3.2. The Improvement of Dry Eye Parameters after Treatment

**Figure 3:**
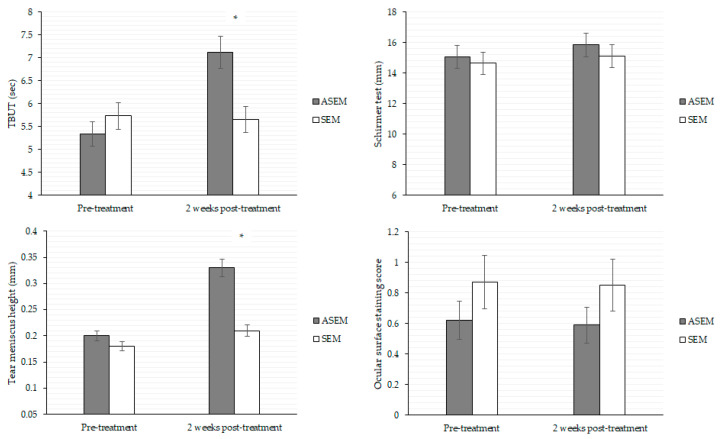
Objective dry eye indexes between the ASEM group and SEM groups after the 2-week treatment. ASEM: acupuncture steam-warming eye mask, N: number, SEM: steam-warming eye mask TBUT: tear break-up time. * denotes significant difference between the two groups (*p* < 0.05).

After the 2-week treatment, the TBUT (7.12 versus 5.65, *p* = 0.011) and tear meniscus height (0.33 versus 0.21, *p* = 0.035) were significantly better in the ASEM group compared to the SEM group, respectively. However, the value of both the Schirmer test scores and ocular surface staining scores showed no significant difference between the two groups (both *p* > 0.05; Figure 3). The alterations in eyelid abnormalities were also comparable between the two groups on Day 15 (all *p* > 0.05), except the ASEM group demonstrated a better improvement of meibum quality in lower eyelid score compared to the SEM group (*p* = 0.014) (Table 2). About the subjective parameter value between the two groups after the 2-week treatment, all symptoms and questionnaire scores improved significantly in the ASEM group (all *p* < 0.05), whereas most of above parameters in the SEM group improved significantly except for the feelings of relaxation, comfortable, and refreshment (both *p* > 0.05; Table 3). 

### 3.3. Comparison of Dry Eye Parameters between Groups

**Figure 4:**
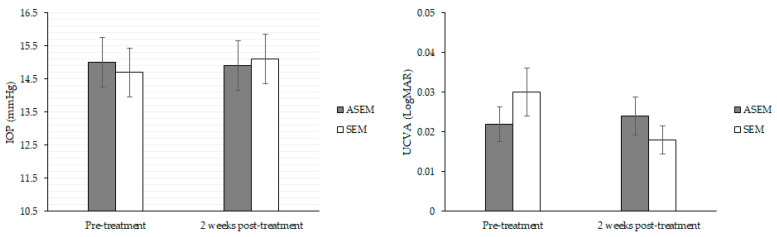
Comparison of safety indexes of treatment between the two groups. ASEM: acupuncture steam-warming eye mask, IOP: intraocular pressure, SEM: steam-warming eye mask, uncorrected visual acuity.

Compared with the SEM group, the ASEM group exhibited a significantly stronger feeling of refreshment (*p* = 0.013), lower sensation of ocular discharge (*p* = 0.031), higher TBUT (*p* = 0.045), better meibomian gland expressibility of both eyelids (both *p* < 0.05), and better meibum quality of both eyelids (both *p* < 0.05) after adjustments for age and sex (Table 4). Other subjective and objective parameters exhibited similar trends of improvement between the two groups (all *p* > 0.05; Table 4). On the other hand, neither IOP nor the UCVA exhibited significant changes after the treatment in either group (both *p* > 0.05; Figure 4).

## 4. Discussion

Our results revealed that subjective DED symptoms had grossly improved in both VDT user groups after treatment. However, compared with the use of the SEM, use of ASEM led to an enhanced feeling of refreshment, decreased sensation of ocular discharge, elevated TBUT, and improved meibum-related indexes of the upper and lower eyelids. Moreover, the safety of ASEM was confirmed by considering the IOP and UCVA.

Acupuncture has been applied as an alternative treatment in several diseases [38]. In traditional Chinese medicine, its main function has been to relieve headache and muscle stiffness. In one study, patients receiving acupuncture experienced less chronic low back pain and fibromyalgia than did the control group [39]. Acupuncture had also been demonstrated to alleviate migraines [40,41]. These effects likely occur because acupuncture can inhibit the NF-kB pathway, which is involved in inflammation [42], as well as the levels of tumor necrosis factor and interleukin [43]. Acupuncture may induce tumor necrosis factor levels which may play an important role in the treatment of dry eye [44]. Applying electroacupuncture to the trigeminal nerve area can stimulate parasympathetic nerve activity and increase cerebral blood flow, which may provide a comforting sensation [45]. All of these pathways can reduce pain and inflammation and alleviate associated symptoms. The application of acupuncture was also reported to decrease ocular irritation and visual analog scores in patients with DED, likely resulting from vagal stimulation and the reduction of chronic inflammation [46]. In another study, the ocular surface disease index and TBUT improved after acupuncture therapy [47]. Moreover, the inflammatory cytokine levels in the tear, including tumor necrosis factor and interleukin, were significantly lower in patients receiving acupuncture therapy than in those receiving artificial tear treatment [48]. Because DED is also an inflammatory disease like chronic pain and rheumatic arthritis [49], a decrement in the levels of inflammatory cytokines can theoretically suppress DED progression and severity. Moreover, acupuncture can induce a “limbic touch” response, which reduces the sensation of pain and thus decreases the subjective symptoms of DED [46]. Because VDT users tend to have a higher prevalence of DED [2,13], the addition of acupuncture to other forms of treatment, such as eyelid warming, may have superior efficacy than monotherapy. This is at least partially supported by the findings of our study.

According to our findings, the ASEM contributed to a stronger feeling of refreshment, lower ocular discharge sensation, higher TBUT and better meibum status than the SEM device without such a function. To the best of our knowledge, this is the first study to demonstrate the effectiveness of combined eyelid-warming mask use and acupuncture on DED in VDT users. We adjusted for age and sex in the generalized linear mixed model, as both are risk factors for DED [50]. Consequently, the ASEM device with acupuncture function may be an independent protective factor for both the objective and subjective DED-related parameters in VDT users. MGD is an inflammatory eyelid disease that causes poor meibum secretion [51]. High interleukin levels have been reported in patients with severe MGD and can serve as a potential indicator of MGD [52]. Because acupuncture can decrease inflammatory cytokine levels [48], it can also improve meibum secretion, thereby improving both meibomian gland expressibility and quality. This may explain why ASEM was better than SEM monotherapy. The TBUT indicates the stability of tear film, which could be affected by the inflammation of ocular surface [49,50]. Our study supports the theory that acupuncture is associated with improvement in the TBUT, as seen with improvement from 5 to 7 s after just 2 weeks of therapy with ASEM compared to no significant change in the SEM group. This combination therapy also decreased the ocular discharge sensation, possibly because the acupuncture-induced reduction of inflammation decreased discharge formation, and the associated pain relief function may diminish the “unpleasant sensation” resulting from ocular discharge. Pain relief may also have contributed to the improvement in the subjective feeling of “refreshment” in our study. By contrast, several objective signs, including Schirmer test scores, tear meniscus height, and ocular surface staining score, did not significantly improve in the ASEM group compared with the SEM group. We speculate that the treatment period of our study was not adequate to affect the objective parameters. Additionally, the patients continued to use VDT for more than 6 h daily during the 2 weeks of treatment because most of them used VDT due to their occupation. Also, they did not have any other change in behavior that might have affected the results according to the inquiry in medical records. As a consequence, further study with longer durations of acupuncture therapy is needed to investigate the exact mechanism of ASEM on improving DED symptoms compared to SEM.

Concerning the effectiveness of the SEM itself, nearly all subjective DED symptoms, including the CVSQ and DEQS scores, were noted to be significantly improved after 2 weeks whether the acupuncture function was used or not. A previous study also reported improvement in the subjective symptoms of DED after 3 weeks of treatment with a SEM [53], consistent with our results. In our previous study, the QoL of VDT users with DED improved significantly after 2 weeks of SEM treatment [28]. The SEM device used in our previous study was the same as that used in the SEM group in the current study, which implies the satisfactory effect of this SEM device in different patient populations and time periods. In the present study, use of the SEM without acupuncture did not improve the feelings of relaxation, comfort, or refreshment, suggesting that the addition of acupuncture can more effectively relieve subjective DED symptoms. Regarding the objective parameters in both the SEM population and the ASEM population, the TBUT, Schirmer test, tear meniscus height, and ocular surface staining score were numerically improved in both groups after 2 weeks of treatment, but the SEM group showed a lower degree of improvement compared to the ASEM group, especially for TBUT and tear meniscus height. Tear meniscus height reflects tear volume, and the moist heat released by the SEM may not have affected the tear volume adequately. Moreover, the lack of acupuncture-related anti-inflammatory effect may cause the slower improvement of TBUT in the SEM group. In short, the lack of significant improvements in the objective parameters in the SEM group probably indicates that the 2-week treatment interval with SEM was not enough to improve these parameters in VDT users, who are under greater DED stress than the general population [3].

Certain complications of acupuncture had been reported [54], such as bruising or bleeding after acupuncture treatment on the lower back [55]. More severe complications include pneumothorax and hemothorax, which can cause death [54,56,57]. Although ophthalmic complications of acupuncture are rare, penetrating eye injury leading to proliferative vitreoretinopathy has been reported [58]. In our study, no posttreatment complications were reported by our participants, and posttreatment IOP and UCVA were comparable with the pretreatment values. This may be partly because the acupuncture function of the ASEM device used in our study was noninvasive—it did not really “puncture” the periocular area but only massaged the three acupoints. Given that this function improved some subjective DED parameters in the ASEM group compared with the SEM group, the ASEM may be both safe and efficacious in the management of DED.

This study has some limitations. First, the 2-week treatment interval may be too short to accurately estimate therapeutic effectiveness. Second, the small total sample size (N = 40) likely diminished the statistical power and affected the reliability of the results, and the variability of patient characteristics including the sex and MGD degrees caused similar effects on our results. Because we collected patients in previous years, collecting new patient may cause a significant change and disturbance of the time of treatment. Third, the patients could use the SEM or ASEM at any time at home; therefore, we could not obtain the exact frequency of SEM and ASEM application in each participant, and the massage acupuncture was also performed by the patients themselves, which may be less accurate. According to the medical records, all the patients reached the 20 min daily usage of ASEM/SEM, but the exact time of ASEM usage (20 min or more) cannot be decided; thus, the effectiveness of ASEM cannot be accessed. Also, because we only recorded the parameters in this study for 2 weeks, an analysis with a longer follow-up period with the same parameters cannot be conducted, and the participants were not blinded as they could feel and see the acupuncture points. All these points may have influenced the therapeutic outcomes. Finally, all the patients were Taiwanese and this is a single-center study, thereby inhibiting the generalizability of our findings.

## 5. Conclusions

In conclusion, the application of the ASEM was associated with a stronger feeling of refreshment, lower ocular discharge sensation, better tear film stability, and better meibum status than SEM in VDT users. Further, 2 weeks of SEM use, regardless of the presence of acupuncture function, increased most of the subjective DED-related symptoms. These findings imply that the use of the ASEM is beneficial for VDT users with prominent subjective DED symptoms, lower TBUT and MGD. Future large prospective studies should investigate the long-term effects of the ASEM for DED in VDT users, and also the effect of ASEM combined with intense pulse light therapy, hyaluronic acid-contained artificial tear or autologous serum treatment.

## Figures and Tables

**Table 1:** Baseline characters of study population.

Characters	ASEM Group (N = 20)	SEM Group (N = 20)	*p* Value
Age	34.17 ± 10.23	35.22 ± 11.41	0.572
Sex (male to female)	6:14	4:16	0.915
TBUT (s, Mean ± SD)	5.34 ± 2.56	5.73 ± 2.37	0.933
Shirmer test value (mm, Mean ± SD)	15.07 ± 9.12	14.65 ± 9.04	0.892
Tear Meniscus Height (mm, Mean ± SD)	0.20 ± 0.07	0.18 ± 0.09	0.084
Ocular surface staining score (Mean ± SD)	0.62 ± 1.07	0.87 ± 1.39	0.580
0–2	17 (85.00%)	14 (70.00%)	
3–6	3 (15.00%)	6 (30.00)	
Meibum expressibility score (mean ± SD)	0.62 ± 0.65	0.64 ± 0.80	0.826
Grade 0	10 (50.00%)	11 (55.00%)	
Grade 1	8 (40.00%)	6 (30.00%)	
Grade 2	2 (10.00%)	2 (10.00%)	
Grade 3	0 (0.00%)	1 (5.00%)	
Meibum quality score (mean ± SD)	1.01 ± 0.82	1.28 ± 0.73	0.484
Grade 0	7 (35.00%)	2 (10.00%)	
Grade 1	8 (40.00%)	13 (65.00%)	
Grade 2	4 (20.00%)	4 (20.00%)	
Grade 3	1 (5.00%)	1 (5.00%)	

ASEM: acupuncture steam-warming eye mask, N: number, SD: standard deviation, SEM: steam-warming eye mask, TBUT: tear break-up time.

**Table 2:** Change in eyelid and ocular abnormalities in the two groups after treatment.

Parameters		ASEM Group (N = 20)	SEM Group (N = 20)	*p* Value
Meibomian gland expressibility				
Upper				0.114
	Improved	8 (40.00%)	3 (15.00%)	
	No change	12 (60.00%)	14 (70.00%)	
	Worsened	0 (0.00%)	3 (15.00%)	
Lower				0.076
	Improved	7 (35.00%)	3 (15.00%)	
	No change	13 (65.00%)	15 (75.00%)	
	Worsened	0 (0.00%)	2 (10.00%)	
Meibum quality				
Upper				0.059
	Improved	8 (40.00%)	6 (30.00%)	
	No change	9 (45.00%)	7 (35.00%)	
	Worsened	3 (15.00%)	7 (35.00%)	
Lower				0.014 *
	Improved	7 (35.00%)	2 (10.00%)	
	No change	12 (60.00%)	10 (50.00%)	
	Worsened	1 (5.00%)	8 (40.00%)	

ASEM: acupuncture steam-warming eye mask, N: number, SEM: steam-warming eye mask. * denotes significant intergroup difference between the two groups (*p* < 0.05).

**Table 3:** Subjective dry eye symptoms in the two groups before and after treatment.

Symptoms	ASEM Group (N = 20)		SEM Group (N = 20)	
	Baseline	2 Weeks	Baseline	2 Weeks
Tired eyes	52.45 ± 25.52	29.14 ± 20.36 *	55.46 ± 18.17	31.66 ± 18.58 *
Dry eyes	49.32 ± 26.42	24.00 ± 21.35 *	52.24 ± 17.38	30.36 ± 21.42 *
Ocular discomfort	39.12 ± 26.07	15.24 ± 14.87 *	44.95 ± 18.20	23.96 ± 18.34 *
Blurred vision	26.45 ± 24.17	13.10 ± 19.01 *	33.62 ± 23.56	22.88 ± 20.50 *
Gritty eyes	16.95 ± 16.54	8.75 ± 10.26 *	32.25 ± 21.39	22.67 ± 21.05 *
Relaxing feeling	62.51 ± 21.73	78.49 ± 16.50 *	61.53 ± 17.78	64.16 ± 22.37
Comfortable feeling	65.13 ± 22.29	76.35 ± 17.12 *	56.88 ± 16.45	63.11 ± 17.02
Refreshing feeling	57.86 ± 20.44	74.97 ± 18.53 *	60.39 ± 15.28	61.07 ± 20.62
Computer visual syndrome	10.07 ± 3.02	5.48 ± 4.65 *	11.42 ± 4.04	7.84 ± 4.83 *
Dry eye questionnaire score	29.35 ± 14.27	15.38 ± 10.22 *	31.27 ± 13.35	19.21 ± 13.98 *

ASEM: acupuncture steam-warming eye mask, N: number, SEM: steam-warming eye mask. * denotes significant intergroup difference before and after treatment (*p* < 0.05).

**Table 4:** Effect of eye-warming mask plus acupuncture on the improvement of dry eye parameters.

Parameter (Reference: SEM Group)	Estimate	Standard Error	t	*p* Value
Tired eyes	0.49	7.2	0.06	0.926
Dry eyes	−3.25	6.5	−0.53	0.641
Ocular discomfort	−2.11	6.3	−0.35	0.753
Blurred vision	−2.19	5.3	−0.42	0.656
Gritty eyes	1.71	4.5	0.32	0.789
Relaxing feeling	8.72	5.7	1.74	0.101
Comfortable feeling	−0.91	5.2	−0.17	0.835
Refreshing feeling	12.68	5.1	2.74	0.013 *
Computer visual syndrome	−0.18	1.2	−0.22	0.878
Dry eye questionnaire score	−1.71	1.2	−1.43	0.147
Ocular discharge	−3.27	0.6	−3.63	0.031 *
TBUT	−1.45	0.8	−1.82	0.045 *
Schirmer test	0.82	2.7	0.11	0.386
Tear meniscus height	0.02	0.02	0.40	0.754
Ocular surface staining score	−0.58	0.86	−0.73	0.521
Meibomian gland expressibility				
Upper	−1.76	0.38	−1.59	0.032 *
Lower	−1.63	1.51	−1.96	0.026 *
Meibum quality				
Upper	−1.71	0.52	−1.83	0.023 *
Lower	−2.65	0.77	−3.38	0.006 *

SEM: steam-warming eye mask, TBUT: tear break-up time. * denotes significant difference with treatment (*p* < 0.05).

## Data Availability

The data used in this study are available from the corresponding author upon reasonable request.
